# P022: Antibiograms of consecutive urinary tract samples in elderly

**DOI:** 10.1186/2047-2994-2-S1-P22

**Published:** 2013-06-20

**Authors:** K Latour, B Jans, S Coenen, R Preal, B Catry

**Affiliations:** 1 Healthcare Associated Infections (NSIH), Brussels, Belgium; 2 Scientific Institute of Public Health, Brussels, Belgium; 3 University of Antwerp, Wilrijk, Brussels, Belgium; 4 Intermutualistic Agency, Brussels, Belgium

## Introduction

Urinary tract infections are the main indication for antimicrobials in elderly.

## Objectives

Despite the treat for resistance dissemination and therapy failure, clinicians are seldom informed on the per patient evolution of antimicrobial resistance in pathogens.

## Methods

Laboratory results were obtained from 13 voluntary diagnostic laboratories (12 hospital-associated) in Belgium during the year 2005. Susceptibility profiles were done by Kirby Bauer disk diffusion according to CLSI. The first two urine samples from patients older than 65 year were included.

## Results

Following organisms were predominantly isolated (N first samples/N second samples): *E. coli* (7188/1654), *E. faecalis* (1282/403), *P. mirabilis* (1230/313), *K. pneumonia* (673/173), *P. aeruginosa* (293/120), *E. aerogenes* (375/203), *S. aureus* (158/54), *M. morganii* (347/89), Group B streptococci (149/31), *C. freundii* complex (101/29). When comparing first versus second samples antibiograms for *E. coli*, a decrease in susceptibility was found for the following antimicrobial agents: cotrimoxazole -6.9%; nitrofurantoin -2.8%, fosfomycin 0.0%; ciprofloxacin -10.8%; cefuroxime -5.6%; amoxicillin-clavulanic acid -5.6%; ampicillin -10.5%. For *E. faecalis*, marked decreases were found for nitrofurantoin -2.4%; fosfomycin -2.2%; -ciprofloxacin -10.3%; and only mild decreases for amoxicillin-clavulanic acid 0.0%; and ampicillin -1.2%. For *K. pneumoniae* decreases were in the range of -2.9 to -4.1% for cotrimoxazole, ciprofloxacine, cefuroxime and amoxicillin-clavulanic acid, and was -12.4% for nitrofurantoin. For *S. aureus* and *C. freundii* no decrease (<-0.1%) was seen for nitrofurantoin and fosfomycin. For *E. aerogenes*, decreases of -18 and -12.5% were found for cotrimoxazole and fosfomycin, respectively. *M. morganii* showed in consecutive samples less susceptibility for cotrimoxazole (-16.2%), fosfomycine (-13.0%) and ciprofloxacin (-10.5%), while only a marginal decrease was found for nitrofurantoin (-0.5%).

## Conclusion

The resistance selection influence of consecutive samples depends on the antibiotic-bacterium combinations, and thus might be taken into account when empiric therapy guidelines for urinary tract infections in elderly are reviewed.

## Disclosure of interest

None declared

